# Post-infection symptoms up to 24 months after COVID-19: a matched cohort study in Berlin, Germany

**DOI:** 10.3389/fpubh.2025.1513664

**Published:** 2025-03-12

**Authors:** Anne Meierkord, Daniel Schulze, Maximilian Gertler, Joachim Seybold, Marcus A. Mall, Tobias Kurth, Frank P. Mockenhaupt, Stefanie Theuring

**Affiliations:** ^1^Institute of International Health, Charité Center for Global Health, Charité - Universitätsmedizin Berlin, Corporate Member of Freie Universität Berlin and Humboldt-Universität zu Berlin, Berlin, Germany; ^2^Centre for International Health Protection, Robert Koch Institute, Berlin, Germany; ^3^Institute of Biometry and Clinical Epidemiology, Charité - Universitätsmedizin Berlin, Corporate Member of Freie Universität Berlin and Humboldt-Universität zu Berlin, Berlin, Germany; ^4^Medical Directorate, Charité - Universitätsmedizin Berlin, Corporate Member of Freie Universität Berlin and Humboldt-Universität zu Berlin, Berlin, Germany; ^5^Department of Pediatric Respiratory Medicine, Immunology and Critical Care Medicine, Charité – Universitätsmedizin Berlin, Corporate Member of Freie Universität Berlin and Humboldt-Universität zu Berlin, Berlin, Germany; ^6^German Centre for Child and Adolescent Health (DZKJ), partner site Berlin, Berlin, Germany; ^7^German Centre for Lung Research (DZL), Berlin, Germany; ^8^Institute of Public Health, Charité - Universitätsmedizin Berlin, Corporate Member of Freie Universität Berlin and Humboldt-Universität zu Berlin, Berlin, Germany

**Keywords:** long COVID, post-acute sequel of COVID-19, post COVID-19 condition, COVID-19, SARS-CoV-2

## Abstract

**Introduction:**

Long-term health consequences after mild COVID-19 are not well described. Our aim was to estimate their prevalence and describe the time course of signs and symptoms for a period of up to 24 months after SARS-CoV-2 infection.

**Methods:**

We conducted a cohort study matched for age, sex, and test week among individuals who had attended the public COVID-19 test center at Charité—Universitätsmedizin Berlin, Germany. In early 2022, 576 former COVID-19 patients (>95% non-hospitalized) and 302 uninfected individuals responded to a questionnaire on retrospective monthly symptoms since the test date up to 24 months ago.

**Results:**

Symptoms compatible with long COVID were present in 42.9% (247/576) of former COVID-19 patients, compared with 21.2% (64/302) in the uninfected group. In former patients, unadjusted odds ratios (OR) were highest for disturbed taste/smell (OR 9.1 [95% CI: 4.0–21.1]), memory difficulties (OR 5.1 [95% CI: 2.9–8.9]), and shortness of breath at rest (OR 4.5 [95% CI: 1.9–10.6]). In most former COVID-19 patients, symptoms occurred in one coherent period and resolved after a median of 6.5 months, while taste/smell disturbance and neurological/cognitive symptoms showed longer times until recovery. Factors associated with long COVID-compatible symptoms included hospitalization, symptomatic COVID-19 infection, low household income and female sex.

**Conclusion:**

Post-infection symptoms in mild COVID-19 patients mostly persist for about half a year, but sometimes longer. Among uninfected individuals who never experienced COVID-19, 21.2% also reported long COVID-compatible symptoms. The current long COVID definition might require revision to prevent misclassification and over-reporting, and to improve diagnosis and prevalence estimates.

## 1 Introduction

The COVID-19 pandemic has led to a devastating number of excess deaths worldwide (1). Furthermore, a significant number of people suffer from long-term health consequences following SARS-CoV-2 infection, commonly referred to as long COVID, post COVID-19 condition, or post-acute COVID-19. This multisystemic illness can cause significant disability and poor quality of life, and it is an economic and public health concern (2). Prevalence estimates vary widely, but a recent meta-analysis reported that at least 45% of COVID-19 survivors went on to experience at least one unresolved symptom (mean follow-up 126 days) (3). Factors identified to be associated with an increased risk of developing long COVID include female sex, older age, higher body mass index, smoking, pre-existing comorbidities and previous hospitalization with COVID-19, while vaccination against COVID-19 with two doses seems to lower the risk compared to no vaccination (4). Long COVID has been defined in several ways (5). The World Health Organization defines it as a condition which “occurs in individuals with a history of probable or confirmed SARS-CoV-2 infection, usually 3 months from the onset, with symptoms that last for at least 2 months and cannot be explained by an alternative diagnosis” (6). Long COVID patients may experience a huge variety of symptoms across multiple organ systems, with fatigue, shortness of breath and cognitive dysfunction among the most common ones (7–9). Myalgic encephalomyelitis/chronic fatigue syndrome and similar chronic conditions have been observed following infections with other common viruses, such as Epstein–Barr virus and cytomegaloviruses (10), irrespective of subtle differences in symptom presentation and underlying physiology (11).

Available data suggest that long COVID typically improves over time, yet it remains a substantial burden for affected individuals, on health systems and the workforce (12). In Germany alone, long COVID has been estimated to have caused 5.7 billion euros in economic losses in 2021 (gross value-added loss) (13). Broadly effective treatments are still lacking, and many newer treatment options remain unexplored (7). Some studies suggest that long COVID persists for 24 months after hospitalization due to COVID-19 (14, 15). However, there is still limited data on the actual long-term consequences of COVID-19 in terms of individual health issues and including appropriate referent individuals, and specifically for patients with mild COVID-19. This limits the understanding of the features of long COVID in different populations, which, in turn, impairs opportunities for management and treatment. We aimed to overcome these deficiencies in a matched cohort study in Berlin, Germany, with an assessment of self-reported signs and symptoms up to 24 months after SARS-CoV-2 infection in predominantly non-hospitalized individuals. Our objectives were to estimate the prevalence and to describe the duration of signs and symptoms compatible with long COVID as well as factors associated with that condition.

## 2 Methods

We conducted a matched cohort study among individuals who attended the COVID-19 test center open to the public at Charité—Universitätsmedizin Berlin between March 2020 and June 2021. This facility was operative during that time, and it was the first such site offering SARS-CoV-2 PCR testing in Berlin for individuals showing suggestive symptoms or having had contact to a positively tested person.

All individuals who tested positive between March 2020 and June 2021 were eligible. Recruitment and data collection took place between December 2021 and June 2022. We contacted them by postal mail, including a study information letter, informed consent form, and questionnaire. For each filled-in questionnaire we received, we contacted negatively tested individuals, matched according to sex, age (10-year groups), and calendar week of their test (+/− maximum 2 weeks). We excluded negatively tested individuals if they had an indication or strong presumption of a SARS-CoV-2 infection before or after their test at the COVID-19 test center of Charité—Universitätsmedizin Berlin, based on the reporting of one of the following events in their questionnaire: a previous or subsequent positive SARS-CoV-2 test result, a positive SARS-CoV-2 antibody test, flu-like symptoms for several days with anosmia or ageusia, or flu-like symptoms after contact to a SARS-CoV-2 positive tested person. As an incentive to participate in the study, three tablets were raffled among all participants who returned a filled-in questionnaire. We also tried to increase study participation by sending follow-up letters to those individuals who did not reply the first time. In addition, we contacted persons who had been PCR-tested as participants of the Berlin Corona School Study (BECOSS) (16) following the same scheme.

### 2.1 Participants

**Figure 1:**
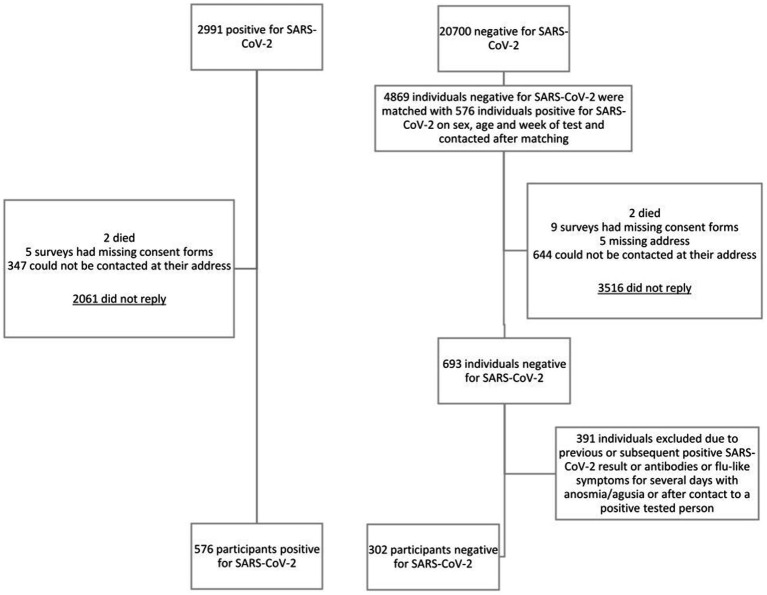
Flowchart of people invited to participate in the study.

Between March 2020 and June 2021, 2,991 individuals tested positive for SARS-CoV-2, and 20,700 tested negative in a single presentation. Individuals tested positive before Omicron became the most prevalent SARS-CoV-2 variant in Germany in January 2022 (17). Among SARS-CoV-2 positives, 68.9% of invitees did not reply, 11.6% could not be reached via their mail address, 0.2% lacked informed consent, and two died. Eventually, 576 SARS-CoV-2 positives were included in the final analysis (Figure 1). We contacted 4,869 SARS-CoV-2 uninfected individuals; 72.2% of those invitees did not respond, 13.2% could not be reached via their mail address, 0.2% lacked informed consent, and two died. 391 (56.4%) of 693 responding potential uninfected individuals were excluded from analysis because of indication or strong presumption of a SARS-CoV-2 infection. This resulted in 302 negatively tested individuals included in our final analysis.

### 2.2 Questionnaire

The questionnaire was designed across a multidisciplinary research group based at Charité—Universitätsmedizin Berlin (Supplementary Questionnaire 1). Data was collected on participants’ sociodemographic variables, SARS-CoV-2 infection status and possible infection since the initial negative test result, severity of SARS-CoV-2 infection including related hospital admission, and any symptoms experienced since the initial test date, i.e., earliest March 2020. We collected data on 23 given symptoms identified as some of the most common long COVID symptoms by previous studies (18, 19). Additionally, respondents had the opportunity to self-report any other symptoms. The questionnaire queried symptom presence for every month between the initial test date and the month of questionnaire completion. Guardians helped filling in questionnaires for children as far as possible. If the child was too young to self-report symptoms guardians reported only symptoms of the child that they could easily observe (e.g., fever, diarrhea or early exhaustion). Most of the study participants were tested positive for SARS-CoV-2 before they received the first vaccination dose, therefore we decided not to include this data into our findings although it was asked on the questionnaire.

### 2.3 Long COVID definition and data analysis

Long COVID was defined according to the current World Health Organization (WHO) definition as the presence of the below symptoms at least 3 months after SARS-CoV-2 infection, lasting for a period of at least two consecutive months (6). Symptoms compatible with long COVID included “exhaustion while resting,” “exhaustion while being minimally active,” “shortness of breath at rest,” “fatigue,” “concentration difficulties,” and “memory difficulties.” We selected these symptoms as the WHO and recent studies identified them as some of the most common and characteristic symptoms in people diagnosed with long COVID (6, 9, 18, 20). Basic characteristics were described by median, range and proportions, as applicable. Monthly symptom prevalence and the course over time between infected and uninfected participants was estimated by comparing the prevalence of reported signs and symptoms. Univariate odds ratios (OR) and 95% confidence intervals (CI) were calculated from cross-tabulation to describe symptoms by infection status and to assess potential factors associated with symptoms compatible with long COVID. All analyses were performed either using SPSS (version: 28.0.1.0.) or R (version: 4.3.1.).

## 3 Results

The percentage of infected and uninfected individuals participating following invitation was 19.3% (576/2991) and 14.2% (693/4869), respectively (Figure 1). Demographic characteristics of infected and uninfected participants are shown in Table 1. Infected participants, as compared to uninfected, were younger, which is likely due to the wide age range of matching. They also had a migration background more frequently and had a tertiary education less frequently.

**Table 1:** Demographic characteristics by infection status.

	Infected, *N* = 576	Uninfected, *N* = 302	Standardized mean difference
	% (n)	% (n)	
Age (years; median, min-max)	37 (1–81)	44 (7–79)	0.35
0–9 years	1.6% (9)	0.3% (1)	
10–19 years	3.1% (18)	1.3% (4)	
20–29 years	22.9% (132)	16.2% (49)	
30–39 years	29.5% (170)	26.2% (79)	
40–49 years	18.1% (104)	18.9% (57)	
50–59 years	16.8% (97)	22.8% (69)	
60–69 years	6.3% (36)	11.6% (35)	
70–79 years	1.6% (9)	2.6% (8)	
80–89 years	0.2% (1)	-	
Female	57.6% (332)	64.6% (195)	0.14
Migration background (at least one parent born outside of Germany)	30.2% (172/571)	21.1% (63/298)	−0.21
Academic Degree	56.5% (321/568)	64.1% (189/295)	0.16
Household income below average (<2000€ netto)	21.8% (108/495)	17.2% (46/267)	−0.12
Single person household	25.7% (147/573)	26.8% (80/298)	0.03

### 3.1 Sign and symptom prevalence

The association toward a profile of symptoms associated with long COVID (i.e., individual symptom lasting at least two consecutive months and occurring for at least 3 months after test result date) was generally higher in infected individuals (Table 2). Most common signs and symptoms (>20% in infected individuals) were fatigue, concentration difficulties, early exhaustion, loss of motivation, depressive mood, memory difficulties, and sleep disturbance. Strongest associations were seen for disturbed taste and/or smell (unadjusted OR 9.1 [95% CI: 4.0–21.1]), memory difficulties (OR 5.1 [95% CI: 2.9–8.9]), shortness of breath at rest (OR 4.5 [95% CI: 1.9–10.6]), concentration difficulties (OR 4.4 [95% CI: 2.8–7.0]), and muscular weakness (OR 4.4 [95% CI: 2.1–9.2]; Table 2). In the uninfected group, most commonly experienced symptoms (>10%) include fatigue, sleep disturbance, headache, depressive mood and a runny nose.

**Table 2:** Reported signs and symptoms lasting at least two consecutive months and occurring for at least 3 months after test result date (cumulative prevalence).

	Infected (576)	Uninfected (302)	
	% (n)	% (n)	Univariate odds ratios (95%CI)*
Fatigue	29.7 (171)	15.6 (47)	2.29 (1.60–3.28)
Concentration difficulties	26.6 (153)	7.6 (23)	4.39 (2.76–6.98)
Exhaustion while being minimal active	23.8 (137)	7.3 (22)	3.97 (2.47–6.38)
Loss of motivation	21.9 (126)	8.6 (26)	2.97 (1.90–2.65)
Depressive mood	21.2 (122)	10.6 (32)	2.27 (1.49–3.44)
Memory difficulties	21.0 (121)	5.0 (15)	5.09 (2.92–8.88)
Sleep disturbance	20.7 (119)	13.2 (40)	1.71 (1.16–2.52)
Headache	18.6 (107)	12.6 (38)	1.59 (1.06–2.37)
Issues with sense of taste and/or smell	15.6 (90)	2.0 (6)	9.14 (3.95–21.14)
Anxiety	12.8 (74)	6.6 (20)	2.08 (1.24–3.48)
Exhaustion while resting	12.3 (71)	7.0 (21)	1.88 (1.13–3.13)
Joint pains	11.6 (67)	7.0 (21)	1.76 (1.06–2.94)
Runny nose	10.8 (62)	10.6 (32)	1.02 (0.65–1.60)
Muscular weakness	10.6 (61)	2.6 (8)	4.35 (2.06–9.22)
Heart palpitations	10.4 (60)	6.0 (18)	1.84 (1.06–3.17)
Cough	9.9 (57)	6.3 (19)	1.64 (0.94–2.80)
Loss of hair	9.5 (55)	3.0 (9)	3.44 (1.67–7.06)
Shortness of breath at rest	8.3 (48)	2.0 (6)	4.49 (1.90–10.60)
Sweats	8.0 (46)	3.3 (10)	2.53 (1.26–5.10)
Chest pain	7.8 (45)	2.6 (8)	3.11 (1.45–6.70)
Migraine	7.8 (45)	5.6 (17)	1.42 (0.80–2.53)
Diarrhea	5.2 (30)	2.0 (6)	2.71 (1.12–6.59)
Fever	2.3 (13)	3.6 (11)	0.61 (0.27–1.38)

*CIs were calculated approximating an asymptotic normal distribution. Additionally, study participants self-reported 123 different symptoms in the free text (99 in infected; 24 in uninfected). None of these were mentioned more frequently than any of the symptoms in the table.

For most of the signs and symptoms, the differences between infected and uninfected participants declined notably from month 6 onwards, with prevalence in infected participants approximating uninfected participants (Supplementary Figure 1). However, some signs and symptoms persisted much longer in the infected group, most notably for the complaints of exhaustion while being minimal active, taste/smell disturbance, fatigue, loss of motivation, concentration difficulties, and memory difficulties. Supplementary Figure 1 shows an illustration of the time course (point prevalence in three-monthly steps) of signs and symptoms shown in Table 2 and provides a reference to the acute symptom phase by including the month 0.

### 3.2 Symptoms compatible with long COVID

**Figure 2:**
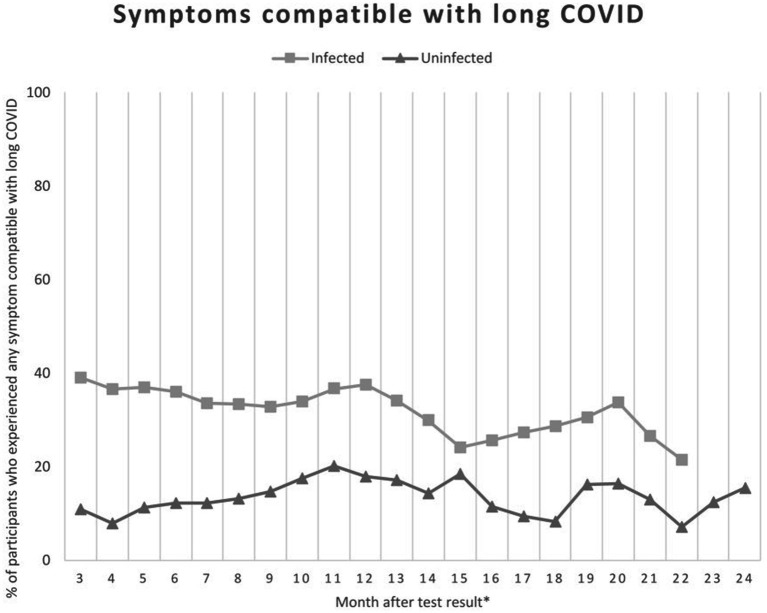
Percentage of participants who experienced any symptom compatible with long COVID following their SARS-CoV-2 test at month 3–24.

Applying the definition as outlined above, the time course of symptoms compatible with long COVID is shown in Figure 2. At 6 months after the initial test, the proportion of individuals reporting symptoms compatible with long COVID was 36.1% (208/576) in former COVID-19 patients and 12.3% (37/302) in uninfected participants, and this figure was 37.6% (172/458 infected) versus 17.9% (46/257 uninfected) and 28.7% (31/108 infected) versus 8.3% (5/60 uninfected) after 12 and 18 months, respectively. Symptoms compatible with long COVID were attributable to 247 (42.9%) of 576 infected participants, including 6 of 22 infected participants below 18 years of age (Table 3). Hence, long COVID was suspected in these individuals. Symptoms compatible with long COVID could also be attributed to 64 (21.2%) of 302 uninfected participants.

**Table 3:** Duration and characterization of symptoms experienced following infection with SARS-CoV-2 in patients with suspected long COVID, *N* = 247.

	Proportion affected by defining condition (%, n)	Symptom subsequent to acute infection (% experiencing symptoms for ≥ 2 consecutive months)	Number of periods of symptom(s) experienced (n, %)	Duration of first period of symptoms (months; median, min-max)	Duration of the second period of symptoms (months; median, min-max)
Fatigue	69.2% (171)	72.7% (125/172)	1: 93.0% (160/172)	8 (2–19)	3 (2–7)
			2: 6.4% (11/172)		
			3: 0.6% (1/172)		
Concentration difficulties	61.9% (153)	83.8% (129/154)	1: 94.2% (145/154)	9 (2–19)	3 (2–6)
			2: 5.2% (8/154)		
			3: 0.6% (1/154)		
Exhaustion while being minimally active	55.5% (137)	71.5% (98/137)	1: 93.4% (128/137)	6 (2–19)	5 (2–8)
			2: 6.6% (9/137)		
Memory difficulties	49.0% (121)	80.2% (97/121)	1: 91.7% (111/121)	7 (2–19)	3.5 (2–10)
			2: 8.3% (10/121)		
Exhaustion while resting	28.7% (71)	66.2% (47/71)	1: 91.5% (65/71)	5 (2–18)	4 (3–7)
			2: 8.5% (6/71)		
Shortness of breath at rest	19.4% (48)	64.6% (31/48)	1: 100% (48/48)	4 (2–18)	N/A
All suspected long COVID*	100% (247) ^**†**^	79.4% (196/247)	1: 26.7% (66/247)	6.5 (2–19)	3.25 (2–10)
			2: 22.3% (55/247)		
			3: 18.2% (45/247)		
			4: 12.1% (30/247)		
			5: 13.8% (34/247)		
			6: 6.9% (17/247)		

*6 out of 22 infected participants are below 18 years of age; ^†^61.9% (153/247) suffered from two or more defining symptoms, 43.3% (107/247) reported that their post-infection symptoms interfered with their daily activities for at least two consecutive months.

The majority of formerly infected, suspected long COVID patients (153/247, 61.9%) suffered from two or more defining symptoms (Table 3). Most patients (>60%) reported symptoms compatible with long COVID in the immediate aftermath of the acute infection. The vast majority (79.4%) experienced one coherent period only, which lasted for an average of 6.5 months (Table 3). In addition, almost half of all formerly infected, suspected long COVID patients (107/247, 43.3%) reported that their post-infection symptoms interfered with their daily activities for at least two consecutive months (Table 3).

### 3.3 Factors associated with symptoms compatible with long COVID

Lastly, we describe factors associated with symptoms compatible with long COVID (Table 4). The univariate ORs were increased in case of hospitalization due to COVID-19 (OR, 4.2; 95% CI: 1.7–10.8), symptomatic infection (OR, 3.4; 95% CI: 1.9–6.3), low household income (OR, 1.8; 95% CI: 1.2–2.7), and female sex (OR, 1.7; 95% CI: 1.2–2.4). Among all infected participants, 4.2% (24/576) reported to have been hospitalized due to COVID-19 (Table 4).

**Table 4:** Sociodemographic and clinical variables of people who tested positive for SARS-CoV-2 and we suspect developed or did not develop long COVID (*N* = 576).

	Suspected long COVID *N* = 247	No long COVID *N* = 329	
	% (n)	% (n)	Univariate odds ratios (95%CI)*
Age (years; median, min-max)	38 (7–81)	36 (1–74)	
Female	64.8% (160)	52.3% (172)	1.68 (1.20–2.36)
Migration background (at least one parent born outside of Germany)	26.6% (65/244)	32.7% (107/327)	0.75 (0.52–1.08)
Academic degree	56.1% (137/244)	56.8% (184/324)	0.97 (0.70–1.36)
Household income below average (<2000€ netto)	27.5% (58/211)	17.6% (50/284)	1.77 (1.16–2.73)
Single person household	29.4% (72/245)	22.9% (75/328)	1.40 (0.96–2.05)
Hospital admission in test month related to COVID-19	7.3% (18)	1.8% (6)	4.23 (1.65–10.83)
Health complaints in test month	94.3% (233)	83.0% (273)	3.41 (1.85–6.29)

*CIs were calculated approximating an asymptotic normal distribution.

## 4 Discussion

Among largely (96%) non-hospitalized patients who tested positive for SARS-CoV-2 up to 24 months ago, 42.9% reported symptoms compatible with long COVID. In most cases, these symptoms occurred subsequent to acute infection in one coherent period, which lasted for a median of 6.5 months but up to a maximum of 19 months. Fatigue, concentration difficulties, and exhaustion at minimal load were the most common symptoms in infected individuals, and the highest odds of reporting disturbed taste and/or smell, memory difficulties, and shortness of breath at rest. Factors associated with suspected long COVID were hospitalization due to COVID-19, symptomatic infection, low household income and female sex.

Approximately 43% of infected participants had symptoms compatible with long COVID, which is exceeding most previously reported long COVID prevalence estimates (20). This figure should be interpreted cautiously, considering that also 21.2% of the uninfected group reported symptoms compatible with long COVID. In other words, the proportion of long COVID suspected individuals is 21.7 percentage points higher for infected than uninfected individuals, a figure corresponding with the WHO long COVID estimate of 10–20% (21). Possible reasons for the high proportion of suspected long COVID in our study include a questionable specificity of the symptoms compatible with long COVID. We used an adapted version of the WHO definition, including six defining symptoms. Previous studies used a range of different definitions and different defining symptoms (5). There is a need for a clear consensus among existing definitions of long COVID (5), as with the reliance of self-reported symptoms and absence of a diagnostic test, many patients struggle to obtain a definitive diagnosis. Hence, long COVID is sometimes falsely dismissed as a psychosomatic condition and vice versa (22). The WHO definition may not capture all the suffering of people with self-reported long COVID, while it is questioned that multiple symptoms are attributed toward this diagnosis (23). Furthermore, this also hinders progress in research harmonization. It is argued that the wide variation of long COVID prevalence estimates is also due to study design heterogeneity (e.g., community, hospitalized), number of assessed symptoms, and methods of assessment (e.g., self-report, healthcare records, clinical investigation) (24). Increased long COVID prevalence has been reported in studies based on self-reported symptoms (24). Given the voluntary nature of our questionnaire-based study, it is possible that symptomatic individuals were more likely to participate than healthy, non-symptomatic individuals, potentially leading to an overestimation of the prevalence of post-infection symptoms. As we lack information on the health status of non-responders, we cannot determine whether our sample is representative of the general German population. Moreover, we did not consider comorbidities or alternative diagnoses and the impact on daily activities for the symptoms, possibly leading to an overestimation of reported symptoms being associated with a SARS-CoV-2 infection, and of the burden of the reported symptoms for an individual. Lastly, the response rate in our study was relatively low, and a comparison of basic characteristics between infected and uninfected participants suggests a selection bias toward older female academics in the uninfected group. It could be argued that the demographic characteristics of the uninfected group are similar to groups at risk of suffering from symptoms of depression (25, 26) and such symptoms potentially overlap with our six defining symptoms of long COVID. The uninfected group might be less healthy than the general population, which could explain the high prevalence of symptoms, but this prevalence is still about 10% higher than national estimates of depressive symptoms in middle-aged females during the first year of the COVID-19 pandemic (25). Research to further explore and understand the high prevalence of symptoms in the uninfected group would be valuable.

Our results align with previous findings that also patients with non-hospitalized SARS-CoV-2 infection exhibit a surplus of subsequent long-term symptoms (27, 28). In the present study, the median symptom duration was 6.5 months, and most symptoms resolved after 1 year. This accords with a recent study on mild COVID-19 in Israel in that most long COVID symptoms resolve within a year from diagnosis (29). Yet, even within half a year, the persisting symptoms as observed in our study, can severely impact quality of life and work ability. Moreover, cognitive dysfunction symptoms seemed to last longer and fade slower than more “physical” conditions. In line with that, cognitive dysfunction symptoms (e.g., difficulties in concentration or memory) occurred in a higher proportion in former COVID-19 patients than what has been estimated in the general population above 50 years of age at 19% (30). This indicates some specificity of this symptom group in long COVID.

Factors associated with suspected long COVID in our study included female sex, low household income, symptomatic infection, and hospitalization due to COVID-19. This is in line with findings among mildly SARS-COV-2 infected populations (4, 27, 28). One reason why females are at increased risk of long COVID might be hormonal patterns perpetuating the hyperinflammation status of acute COVID-19 even after recovery (31, 32). Additionally, stronger IgG antibody responses in females during the acute phase (33) could contribute to persistent symptoms (28). Low income increasing the odds of long COVID has previously been described (34), highlighting the importance of raising awareness of long COVID in this group and ensuring equitable access to rehabilitation and treatment opportunities. Furthermore, it is critical to include marginalized populations, such as migrant populations and low-income households in research studies, as a majority of long COVID research has focused on white, socioeconomically privileged communities (7). Higher prevalence of long COVID among more severe COVID-19 patients is well described (4, 35). However, severe COVID-19 infection is often associated with an Intensive Care Unit (ICU) admission, and ICU survivors are well-known to suffer from post intensive care syndrome (36, 37), which may overlap in its clinical presentation with long COVID. Nevertheless, the importance of long COVID prevention and increased rehabilitation needs are clear among this group at high risk of prolonged health issues. Other associated factors in predominantly mildly affected populations include older age (27), yet younger age has also been described as a risk factor for long COVID in Switzerland (38), reflecting the need for further research on this topic.

Our study found that most symptoms compatible with long COVID presented subsequent to acute infection, underlining the importance of considering this fact in ongoing revisions of a long COVID definition. Our study found that usually symptoms compatible with long COVID presented in one coherent period, however, this is contrasting to the result of other studies showing a fluctuating or relapsing pattern (39, 40). We could not display such “roller coaster” of post-COVID symptoms with our data, likely because we asked for symptom presence by each month. This highlights the importance of including finer assessment intervals than months in future research, particularly as the follow-up time increases of studies assessing long COVID.

Our study has several strengths. We have a relatively long follow-up period, allowing us to analyze the course of progression of long COVID symptoms over time. We used a matched, uninfected group as comparison, which allowed us to account for environmental factors potentially affecting the development of signs and symptoms (e.g., pandemic interventions such as lockdowns). Although our sample may not be fully representative, the diversity of participants still supports the potential relevance of our findings at the population level. Our study should be interpreted in the context of its limitations. First, our study used self-reported symptoms for a time period of up to 24 months as a basis of analysis which meant that in some cases, participants had to recall any signs and symptoms for a period of 2 years. Therefore, recall bias may be present. Second, we asked participants to report symptoms monthly even when a symptom was only experienced for 1 day of the month, possibly allocating too much emphasis and importance to individual symptoms. Third, we might have missed patients with severe forms of post-infection symptoms as they might not have been physically or cognitively able to answer our questionnaire, hence our study population is likely composed of mostly mild to moderate forms of post-infection symptoms.

## 5 Conclusion

While long COVID symptoms often improve over time, with most resolving within 6 months, our findings suggest that symptoms can persist for over a year in some patients, even those with mild infections, potentially posing a long-term social and health burden. Cognitive symptoms, such as concentration and memory difficulties, were particularly persistent. Additionally, symptoms compatible with long COVID were also identified in uninfected individuals, highlighting the challenge of accurately estimating long COVID prevalence. The use of an imprecise definition likely leads to patient misclassification and biases in study findings. Therefore, a more precise definition of long COVID is needed to improve diagnosis on an individual level and estimates on a population level. This would help to harmonize research, identify risk groups and calculate realistic estimates of the burden of long COVID.

## Data Availability

The datasets generated and analyzed during the current study are not publicly available but are available from the corresponding author on reasonable request.
