# Supplementary material for: Post-infection symptoms up to 24 months after COVID-19: a matched cohort study in Berlin, Germany
Source: Front Public Health. 2025 Mar 12;13:1513664. doi: 10.3389/fpubh.2025.1513664 (PMC11937017; doi:10.3389/fpubh.2025.1513664)
Supplement: Supplementary file 1 [file Data_Sheet_1.PDF]

Questionnaire: Berlin Corona Follow-Up Study (BeCoFU)

If children are study participants: Dear parents, please help your child with answering the following questions.

Date (today): ☐☐☐☐☐☐☐☐ (DD/MM/YYYY) **Codenumber (ID):** ……………….(by study team)

1a) Date of birth: ☐☐☐☐☐☐☐☐ (DD/MM/YYYY)

1b) Sex: ☐ Male ☐ Female ☐ divers

1c) Nationality: ☐ German ☐ Turkish ☐ Polish ☐ other:……………………..

1d) Highest form of education:

☐ None ☐ Secondary school (Hauptschulabschluss) ☐ Secondary school (Realschulabschluss)

☐ University-entrance diploma ☐ Apprenticeship ☐ University degree

1e) Current form of employment:

☐ Fulltime ☐ Part-time ☐ Job seeking ☐ Self employed

☐ Unable to work ☐ Retired ☐ Housewife/husband ☐ in education

1f) What is the average <u>monthly</u> income of your <u>whole</u> household?

☐ under 1000€ net ☐ 1001€-2000€ net

☐ 2001€-5000€ net ☐ more than 5000€ net

☐ I do not want to disclose information on this

1g) Were both of your parents born in Germany? ☐ yes, both ☐ no, just one or none

1h) How many people live in your household (including you): ☐ 1 ☐ 2 ☐ 3 ☐ 4 ☐ 5 ☐ ≥ 6

Thereof children (<18 years): ☐ 0 ☐ 1 ☐ 2 ☐ 3 ☐ 4 ☐ ≥ 5

1i) Which languages are mainly spoken in your household? (more than one answer possible)

☐ German ☐ Turkish ☐ Arabic ☐ Russian

☐ English ☐ Polish ☐ Others:…………………………………………..

1j) Have you been vaccinated for COVID-19 yet? ☐ yes ☐ no

If yes: COVID-19 Impfung,

1. Dosis, Date: ☐☐☐☐☐☐☐☐ (DD/MM/YYYY) Vaccine:………………………………………………..

2. Dosis, Date: ☐☐☐☐☐☐☐☐ (DD/MM/YYYY) Vaccine:………………………………………………..

3. Dosis, Date: ☐☐☐☐☐☐☐☐ (DD/MM/YYYY) Vaccine:………………………………………………..

1k) I am happy to be contacted again after 3 months by the study team: ☐ yes ☐ no

2. SARS-CoV-2 Infection status

Have you ever been tested positive for SARS-CoV-2 (PCR Test)? ☐ **no** ☐ **yes**

If yes: When was the first time you were tested positive? ________________________________ (DD/MM/YYYY; Date of sampling)

If no: Did you experience any of the following **since March 2020**? (more than one answer possible)

- Cold symptoms with loss of taste/smell lasting several days ☐ no ☐ yes
- Cold symptoms after contact with a person who has been tested positive for SARS-CoV-2 ☐ no ☐ yes
- Positive rapid antigen test ☐ no ☐ yes
- Positive antibody test **(blood test)** ☐ no ☐ yes

3. Symptoms

3a) Please mark below if and in which time period you have been experiencing symptoms and complaints (please use „X“ to mark the month, even when you have only experienced complaints for a few or a single day :

	2020										2021												2022	
	Mar	Apr	May	Jun	Jul	Aug	Sep	Oct	Nov	Dec	Jan	Feb	Mar	Apr	May	Jun	Jul	Aug	Sep	Oct	Nov	Dec	Jan	today
No complaints																								
Symptoms, which did **not** disrupt daily activities																								
Symptoms, which **did** disrupt daily acitivities																								
Complaints, for which **medical** care was seeked																								
In this month I was certified unfit for practice (or partly)																								
In this month I was certified unfit for practice (or partly) due to COVID-19 related symptoms																								
Hospitalisation																								
Hospitalisation due to COVID-19 related symptoms																								

3b) Please mark below if and which symptoms you have experienced or are still experiencing (please use „**X**“ to mark the month, even when you have only experienced complaints for a few or a single day.)

	2020										2021												2022	
	Mar	Apr	May	Jun	Jul	Aug	Sep	Oct	Nov	Dec	Jan	Feb	Mar	Apr	May	Jun	Jul	Aug	Sep	Oct	Nov	Dec	Jan	today
Fever																								
Runny nose																								
Cough																								
Sweats																								
Headache																								
Migrane																								
Loss of power in muscles																								
Joint pains																								
Exhaustion while resting																								
Exhaustion while being minimal active																								
Shortness of breath at rest																								
Issues with sense of taste and/or smell																								
Tiredness																								
Loss of motivation																								
Chest pain																								
Sleep disturbance																								
Concentration difficulties																								
Anxiety																								
Difficulties to remember things																								
Depressive mood																								
Heart palpitations																								
Loss of hair																								
Diarrhoea																								
Other: ………………………………																								
Other: ………………………………																								

2c) Do you currently receive medical care for any of the above mentioned symptoms? ☐ **no** ☐ **yes**

4. Medical care infrastructure (only for people who tested positive in 2020 or 2021)

Please answer the following questions which ask about your personal situation and medical care infrastructure after you tested positive for SARS-CoV-2 (Please remember that all this data will be analysed without connection to you as an individual (pseudonymized) and are medically confidential data)

4a) For the time that you spent in self-isolation at home after you tested positive for SARS-CoV-2: Did you experience the desire to access medical care?

☐ No
☐ Yes, once
☐ Yes, twice or more

4b) If yes: Which places did you try contacting <u>first</u>? (Please just select one response)

☐ GP (Call or Video)
☐ Called KV-Emergency care/KV-Hotline (Tel. 116117)
☐ Called COVID-19-Hotline of the senat of Berlin (Tel. 9028282828)
☐ Called Ambulance (Tel. 112)
☐ Community nursing care contacted
☐ Emergency button at home pressed (Hausnotruf)
☐ I did not phone anyone, went straight to the doctors practice
☐ I did not phone anyone, went straight to A&E
☐ I did not do anything

4c) If you tried getting help via phone call, how successful was that phone call? (More than one answer possible)

☐ No one answered.
☐ Telephone consultation. My questions/concerns were addressed, no further consultation needed.
☐ Telephone consultation resulted in a home visit (by medical staff from GP practice or KV-emergency service)
☐ Telephone consultation resulted in a visit of a minor injuries unit, A&E or hospital.

4d) Have you ever called an ambulance because you could not receive any other help/advice?

☐ No ☐ Yes

4e) Have you left self-isolation once or more than once (before the end of official isolation period) to access medical care?

☐ No ☐ Yes ☐ I do not want to disclose any information

4f) Have you left self-isolation once or more than once (before the end of official isolation period) to go shopping or similar?

☐ No ☐ Yes ☐ I do not want to disclose any information

4g) How successful were you at practicing self-isolation at home after your positive test result (separate room to sleep, separate dinner room, contact to family members/roommates in the same room with a medical mask)?

☐ I live by myself
☐ Worked well (for example separate room to sleep, contact always with a mask)
☐ Limited (for example only individual events where there was contact to another person without a mask)
☐ Did not work well (for example separate room but daily contact to household members without a mask)
☐ Not possible (for example no separate room, care provided for children and/or household members)
