# Supplementary material for: Post-infection symptoms up to 24 months after COVID-19: a matched cohort study in Berlin, Germany
Source: Front Public Health. 2025 Mar 12;13:1513664. doi: 10.3389/fpubh.2025.1513664 (PMC11937017; doi:10.3389/fpubh.2025.1513664)

Supplementary Figure 1. Percentage of participants experiencing any signs or symptoms following their SARS-CoV-2 test at 0 (N=878), 3 (N=878), 6 (N=878), 9 (N=858), 12 (N=715), 15 (N=276) and 18 (N=168) months.*
*For clarity and improved overview, 3-month intervals are shown

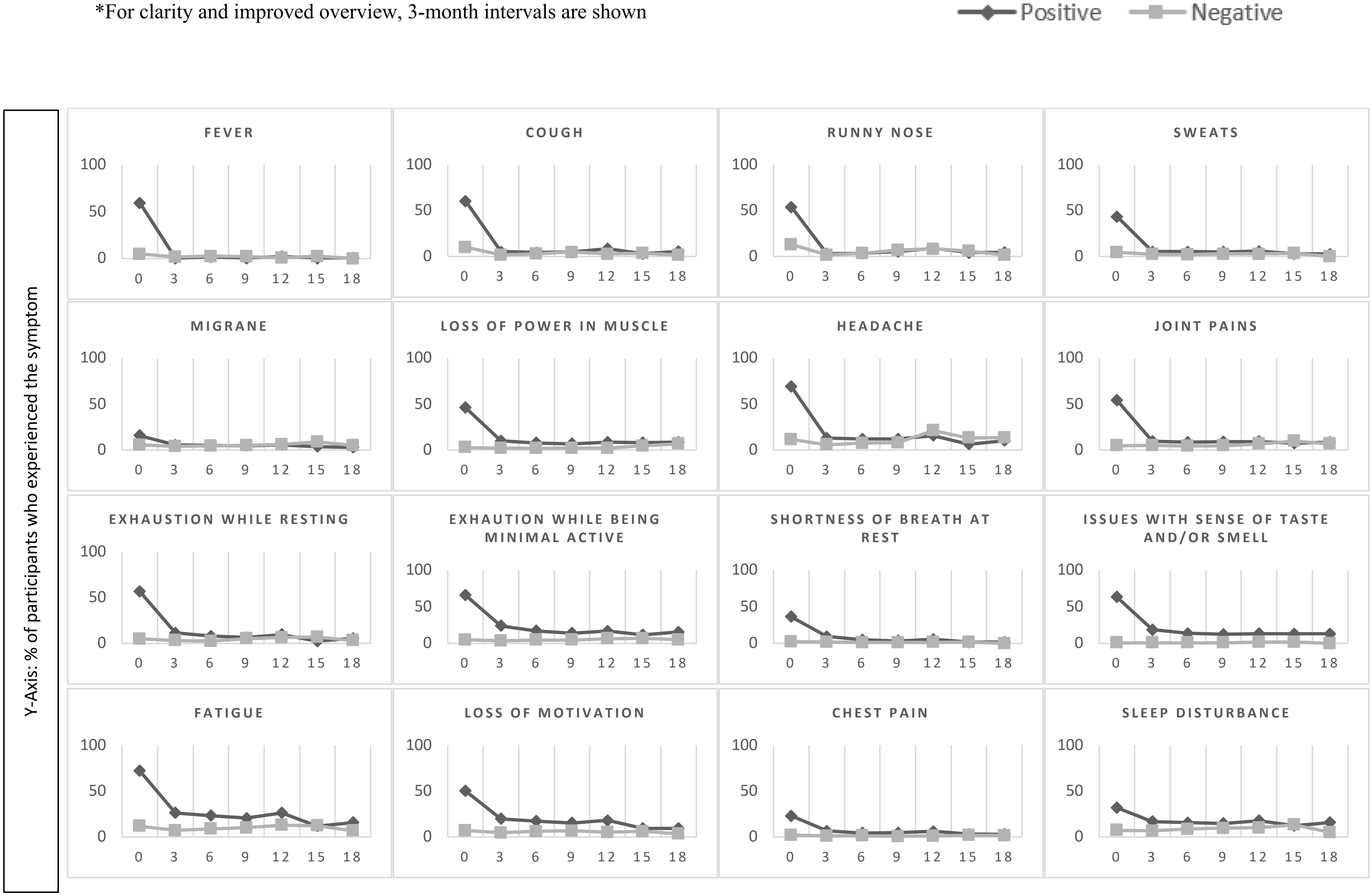

Supplementary Figure 1. Percentage of participants experiencing any signs or symptoms following their SARS-CoV-2 test at 0 (N=878), 3 (N=878), 6 (N=878), 9 (N=858), 12 (N=715), 15 (N=276) and 18 (N=168) months.*
*For clarity and improved overview, 3-month intervals are shown

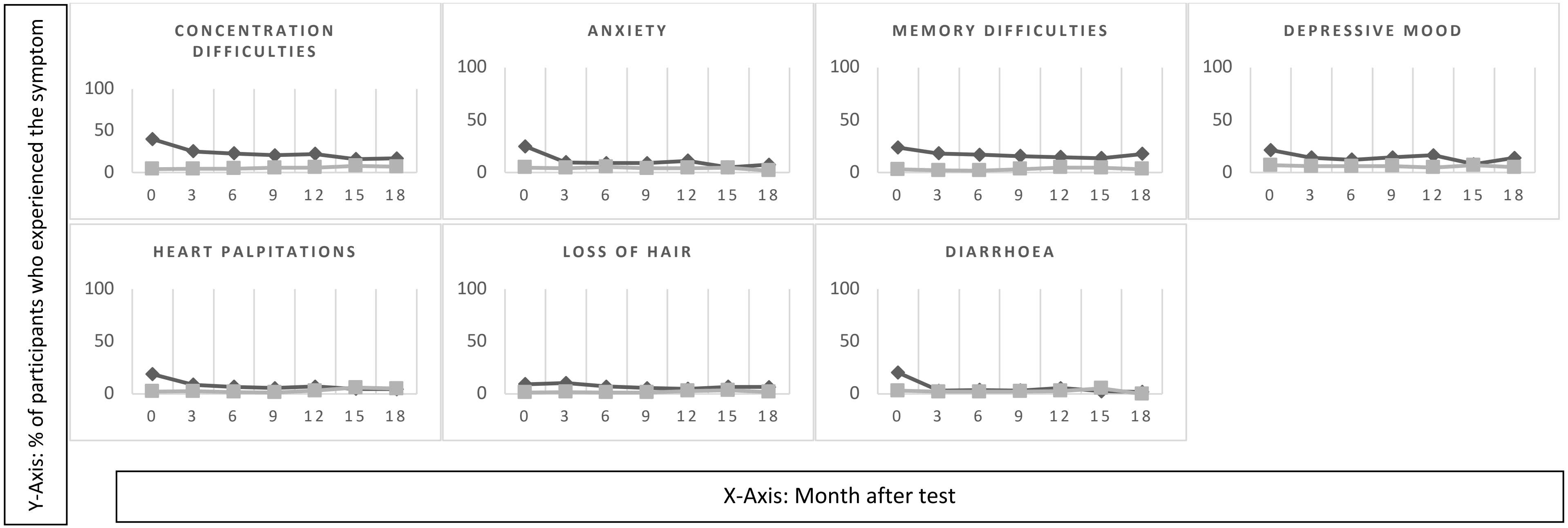

Supplement: Supplementary file 2 [file Data_Sheet_2.PDF]
